# Hybrid liquid biphasic system for cell disruption and simultaneous lipid extraction from microalgae *Chlorella sorokiniana* CY-1 for biofuel production

**DOI:** 10.1186/s13068-019-1591-8

**Published:** 2019-10-25

**Authors:** Guo Yong Yew, Kit Wayne Chew, Marlinda Abdul Malek, Yeek-Chia Ho, Wei-Hsin Chen, Tau Chuan Ling, Pau Loke Show

**Affiliations:** 1 Department of Chemical and Environmental Engineering, Faculty of Science and Engineering, University of Nottingham Malaysia, Jalan Broga, 43500 Semenyih, Selangor Malaysia; 2 School of Mathematical Sciences, Faculty of Science and Engineering, University of Nottingham Malaysia, Jalan Broga, 43500 Semenyih, Selangor Malaysia; 3 Institute of Sustainable Energy (ISE), University Tenaga National, 43000 Kajang, Selangor Malaysia; 4 Civil and Environmental Engineering Department, Universiti Teknologi PETRONAS, 32610 Seri Iskandar, Perak Darul Ridzuan, Malaysia; 5 Centre for Urban Resource Sustainability, Institute of Self-Sustainable Building, Universiti Teknologi PETRONAS, 32610 Seri Iskandar, Perak Darul Ridzuan, Malaysia; 6 Department of Aeronautics and Astronautics, National Cheng Kung University, Tainan City, Taiwan; 7 Institute of Biological Sciences, Faculty of Science, University of Malaya, 50603 Kuala Lumpur, Malaysia

**Keywords:** Radical, Lipid, Microalgae, Biofuel, Extraction

## Abstract

**Background:**

The extraction of lipids from microalgae requires a pretreatment process to break the cell wall and subsequent extraction processes to obtain the lipids for biofuels production. The multistep operation tends to incur high costs and are energy intensive due to longer process operations. This research work applies the combination of radicals from hydrogen peroxide with an organic solvent as a chemical pretreatment method for disrupting the cell wall of microalgae and simultaneously extracting lipids from the biomass in a one-step biphasic solution.

**Result:**

Several parameters which can affect the biphasic system were analyzed: contact time, volume of solvent, volume ratio, type of organic solvent, biomass amount and concentration of solvents, to extract the highest amount of lipids from microalgae. The results were optimized and up to 83.5% of lipid recovery yield and 94.6% of enhancement was successfully achieved. The results obtain from GC-FID were similar to the analysis of triglyceride lipid standard.

**Conclusion:**

The profound hybrid biphasic system shows great potential to radically disrupt the cell wall of microalgae and instantaneously extract lipids in a single-step approach. The lipids extracted were tested to for its comparability to biodiesel performance.

## Introduction

Microalgae consists of high lipid content and is very potential as a sustainable feedstock for commercial biofuel production. The lipid content in microalgae constitutes of more than 50% of the total composition in the biomass [1, 2]. They are listed as a third-generation of biomass which can convert carbon dioxide into lipids and biofuels through transesterification. The productivity of microalgae oil in liters per hectare of land is higher compared to palm oil and sunflower oil by more than 16-fold [3, 4]. Furthermore, microalgae can grow in abundance and emerge as a potential source for lipid extraction for biofuel generation to replace petroleum fuel [5]. Lipids are a class of organic compounds which comprises of fatty acids and glycerol in its natural form, commonly known as triglyceride. The microalgae cell contains various types of lipid, however, the lipid favored for biofuel production are neutral lipids. Phospholipids and glycolipids are polar lipids which belongs to the category of structural lipids mainly used for construction of cell wall membranes, such as being integrated with fiber and polysaccharide in the raw molecular compound in the cell wall membrane [6]. The extraction technique for lipid extraction commonly involves the use of organic solvents to dissolve lipids and further purification of the lipid content by evaporating the solvents [7]. The lipids from microalgae could be processed to produce cosmetic products, biofuel, nutraceuticals, and synthetic polymer [2, 8–10].

The conventional solvent extraction techniques using alcohol and organic solvents by Folch method or Bligh and Dyer method is a common practice for lipid extraction in a laboratory scale [11, 12]. The alcohol solvent is prone to attracting polar compounds as it contains hydroxyl group molecules (OH), while organic solvent is categorized as non-polar solvent which can dissolve lipids in the solution. The molecular structure of every alcohol contains similar carbon chain with non-polar characteristic due to the carbon and hydrogen both consisting of low electronegativity difference, and OH group with polar properties attached at the tail of the chains [13, 14]. Thus, alcohol has been categorized as a polar group of solvent due to the presence of hydroxyl functional group in the chains. Typical solvent extraction uses different type of solvents in the immiscible phase, where the distribution of ions and particle may occur according to the compatibility of the types of solvent to attract or release ions in the solution [15]. The conventional alcohol and organic solvent extraction does not create a significant cell disruption mechanism for the microalgae biomass. Pre-treatment processes such as mechanical or chemical method are needed to induce the cell wall membrane disruption before the lipid compounds may be released and readily dissolve into the organic solvent [16].

Current technologies used for mechanical pre-treatments in microalgae cell disruption includes bead milling, high-pressure homogenization, ultrasonication, microwave, pulse electric field, cavitation and thermal disruption methods [17]. These cell disruption techniques are reviewed intensively for the industrial algae biorefinery processing. The efficiency of mechanical cell disruption methods is based on energy input and duration of the process, where most of these techniques are highly energy intensive and require costly equipment [18, 19]. On the other hand, cell wall could be disrupted by chemical treatments such as acids, bases, and surfactants [20]. Chemical treatments are often associated with concerns on contamination and product quality compromise which is observed for food products extraction such as protein, antioxidant, and others nutrient supplements. The use of chemical treatments provides greater contact surface for liquid cell disruption compared to mechanical methods as the mechanical pretreatments rely on the contact orientation and is mobility restraint. The introduction of peroxyl cell disruption as a chemical treatment layer for enhancing extractability of microalgae lipids can be considered through the possibility of radical formation including peroxidation for the lipids. The biofuel production performance from biomass has also been reported to improve through applying radical cell disruption methods [21].

The oxygen–oxygen bond homolyzes in hydrogen peroxide (HOOH) forming a radical pair, in which the separation of the two hydroxyl radicals (OH) are in symmetry and there are more than 10 kJ/mol of energy needed. The difference of heterolysis and homolyzes of the dissociation components from the molecules are between ions and radicals. Heterolysis is a process of cleaving or loosening of electrons from the original bonded pair of the elements to form ions charge in the solution [22]. The transfer of ion occurs by breaking itself from a neutral molecule and subsequently made into a form of cation and anion from their parent compounds. Homolyzes is a process where the mechanism of radical forming in which the chemical bond dissociates from a molecule and form radical pairs [21, 23]. Each of the fragments obtain equal quantity of the original bonded electron. There is a possibility that applying heat or light source such as UV-ray to the bonds from the solution can trigger the homolytic and heterolytic cleavage process and generate radicals and ions [24].

In this work, a single-step extractive method with radical cell disruption properties for lipids extraction purpose was performed. This hybrid method involves the cell disruption of microalgae using a chemical base liquid mixture with an organic solvent for dissolving the lipids. The mixture of two types of liquids will form a biphasic system and the organic phase containing the lipid was characterized for the lipid concentration. Process parameters such as the concentration of the hydrogen peroxide, type of organic solvent, concentration of the organic solvent, ratio alternation, biomass variation, and volume of solvent, was optimized throughout the experiment to obtain the maximum recovery of lipid from microalgae biomass.

## Materials and methods

### Materials

Hydrogen peroxide (H_2_O_2_), hexane (C_6_H_14_), chloroform (CHCl_3_), acetone (C_3_H_6_O), methanol (CH_3_OH), and sodium chloride (NaCl) were purchased from R&M chemicals (Malaysia). Triglyceride lipids standard mixture were purchased from Sigma Aldrich (USA). All chemicals and solvents used were of analytical grade.

### Microalgae biomass cultivation

*Chlorella sorokiniana* CY-1 strain has been selected in this study due to its high lipid content and the strain was provided by the National Cheng Kung University, Taiwan. *C. sorokiniana* CY-1 was cultivated in a laboratory scale vertical photobioreactor (PBR) as [25] using BG-11 medium containing (g/L): NaNO_3_, 1.5; K_2_HPO_4_, 0.03; MgSO_4_·7H_2_O, 0.075; citric acid, 0.006; Na_2_CO_3_, 2.0; CaCl_2_·2H_2_O, 3.6; Ferric ammonium nitrate, 0.6, EDTA, 0.1; H_3_BO_3_, 2.86; MnCl_2_·4H_2_O, 1.81; ZnSO_4_·7H_2_O, 0.222; Na_2_MoO_4_·2H_2_O, 0.39; CuSO_4_·5H_2_O, 0.079; Co(NO_3_)·6H_2_O, 0.049. The pre-culture and batch culture were both conducted in an indoor PBR system consisting of a 1 L glass vessel. The PBR was aerated with compressed air and an additional 5% of CO_2_ from a tank. The aerated air is filtered through a 0.45 μm air filter before entering the PBR. The photobioreactor was illuminated by external LED light sources with a light intensity of 200 μmol/m^2^/s on both sides of the vessel. The light intensity was determined by using LI-190SA pyranometer sensor (LI-COR, Lincoln USA). The operating temperature was in room condition around 25–27 °C with agitation speed of 300 rpm. Adequate biomass content was cultivated for the whole experiment.

### Determination of nitrate content and microalgae cell concentration

Nitrate content and microalgae cell concentration was measured by spectrophotometric method as mentioned in a previous research [26]. For nitrate content, the supernatant fluid after centrifugation of the microalgae samples was obtained and diluted with deionized water. The nitrate content was measured at optical density of 220 nm using UV–Vis spectrophotometer (UV-1800, Shimadzu). While for microalgae cell concentration, the microalgae samples were measured directly at an optical density 680 nm. Harvesting of microalgae was conducted when the biomass and nitrate level reached a constant value. The approximation of harvesting period was around 14–16 days, where the microalgae culture broth harvested was centrifuged at 7000 rpm for 5 min. The microalgae biomass was freeze dried for 24 h and the resulting microalgae powder was used for experimental extraction studies.

### Hybrid liquid biphasic system with radical cell disruption properties

The hybrid liquid biphasic system (HLBS) was generated by initially adding functionalized hydrogen peroxide (functionalized by exposing hydrogen peroxide to the florescent lamp at intensity of 8 µmol/m^2^/s for initiating the radical propagation) to 20 mg of biomass, followed by the addition of organic solvent for dissolving lipid in a 1:1 volume ratio of mixture. The solution was vortexed for 10 s and left for 1 min to allow the contact of the liquid mixture. Two phases were formed where the upper phase was hexane and lower phase was hydrogen peroxide disruption solvent. The organic solvent phase was separated using a pipette and analyzed for lipids concentration. Lipid triglyceride concentration was optimized at every parameter by utilizing the UV–Vis spectrometer at range of 220 nm–290 nm, in which the exact value for lipid content in hexane and chloroform are at 239 and 223.5 nm, respectively [27–29]. The final optimized lipid was tested through Perkin Elmer GC-FID. The column used was Perkin Elmer Elite-5 (30 m × 0.32 mm × id 0.25 μm) with helium 1.0 mL/min, column oven temperature held at 35 °C for 3 min, increasing to 200 °C with increment of 20 °C/min. The split ratio was 15:1. The concentration of organic solvent (20%) was prepared by diluting with acetone to form a homogeneous phase of solvent. Acetone is stable with oxidant solvent and mixes well with chloroform and hexane (ECHA 2019).

### Total lipid analysis

Total lipid content in the microalgae biomass was determined using the following methods [12, 30]. Approximately 20 mg (± 0.3 mg) of microalgae biomass powder was filled into a 10 mL centrifuge tube. 3 mL of the solvent mixture, chloroform:methanol (2:1, v/v) was added. The contact duration for microalgae biomass in the solution was set at 30 min to allow the biomass to settle down and particulate in the solution. The supernatant lipid mixture was extracted using micropipette and transfered to a separate tube. The solution was heated at 60 °C for 1 h, then washed with 0.9% NaCl solution by 20% volume of the solution mixture. After vortexed, the mixture and the solution will form two phases, where the upper phase will be siphoned out and the lower phase containing lipids was dried by evaporation in a fume hood at room temperature condition for 3 h [31, 32]. The total weight of lipid extract remaining after evaporation was measured (Table 1).

**Table 1:** Operating parameter and conditions

No.	Operating parameter	Initial condition	Variables	Unit
1	Concentration of hydrogen peroxide	5	5, 10, 15, 20, 25, 30	%w/w
2	Type of solvents and concentration	Hexane, 20	Hexane: 20, 40, 60, 80, ~ 85 (Max) Chloroform: 20, 40, 60, 80, ~ 99.5 (Max)	%w/w
3	Contact time	1 min	1, 2, 3, 4	min
4	Biomass amounts	20	20, 30, 40, 50	mg
5	Ratio of solvents—cell disruption solvent (hydrogen peroxide): organic solvent (hexane)	1:1	1:1, 1:2, 1:3, 2:1, 2:3, 3:1, 3:2	–
6	Volume—hydrogen peroxide (*X*), organic solvent (*Y*)	*X*: 1, *Y*: 3	Hydrogen peroxide (X): 1, 2, 3, 4 Organic solvent (Y): 3, 4, 5, 6	mL

### Determination of recovery yield and enhancement value

The recovery yield (*R*, %) of lipids from the total lipid content was represented by Eq. 1 below:

1 $${\text{Recovery}}\ {\text{yield}}, R \left( \% \right) = \frac{\text{Total lipid recovered in organic solvent phase}}{\text{Total lipid content in microalgae biomass}} \times 100\% .$$

The comparison percentage of using organic solvent biphasic compared to using water (without cell disruption solvent) is known as the enhancement value (*E*, Eq. 2). For the non-disruptive solvent extraction, the microalgae biomass was dissolved in distilled water in room temperature followed by the addition of organic solvent. The contact time duration was set as 1 min.

2 $${\text{Enhancement}}, \;E \left( \% \right) = \frac{{A_{i} - H_{\text{ref}} }}{{A_{i} }} \times 100\%$$

where *A*_i_ stands for optimized concentration of lipids, and *H*_ref_ stands for H_2_O with organic solvent (biphasic system without cell disruption properties). The results were analyzed for three repetitive individual samples.

### Chemical reaction chains of radical formation

The potential of using radicals generated by the Fenton reaction to promote cell disruption for microalgae was experimentally tested [21]. Hydrogen peroxide was used without introducing Ferric ion to initiate the radical in the presence of conventional room fluorescent lamp condition. The reaction ID R3–R4 is the lumped reaction with rate constant *k*_*d*_ and *k*_Lp_ respectively, as shown in Table 2.

**Table 2:** Model parameter for estimating the time of hydroxyl radical formation

Symbol	Value	Units	References
*k*_*d*_	5.00 × 10^8^	L mol^−1^ s^−1^	[34]
*k*_*Lp*_	5.44 × 10^7^	L mol^−1^ s^−1^	[21]
*Y*_L/X_	0.596	–	–
*Y*_OH/X_	2.27 × 10^4^	–	[21]

In Table 2, the model parameters for estimating OH* radical concentration were shown. The lipid yield coefficient *Y*_L/X_ (mg_lip_/mg_biomass_) mentioned in [33] was used to anticipate the capability of oxidizing organic compounds in the solution, while the yield coefficient *Y*_OH/X_ depicts the amount of OH* radical needed for rupturing cell wall of microalgae. *k*_*d*_ is the rate constant for Eq. (3) and *k*_Lp_ is the rate constant for Eq. (4).

The OH* radical produced by hydrogen peroxide may react with several compounds released from microalgae. Thus, the cell wall may cause degradation and consequent disruption on the protective cell layer. These reactions are known as a lumped reaction scheme to describe the overall disruption process [21]. The key radical formation steps as reported in Table 3.

**Table 3:** Rate constant and reaction equation

Reaction ID	Reaction symbol and equation	*K*-value	Units	Reference
R5	H_2_O_2_ → 2 OH* (presence of UV)	10^9^–10^10^	L mol^−1^ s^−1^	[35]
R6	OH* + H_2_O_2_ → H_2_O + HO_2_*	3.2 × 10^7^	L mol^−1^ s^−1^	[36]
R7	L* + O_2_ → LO_2_*	3.0–4.6 × 10^8^	L mol^−1^ s^−1^	[21]
R8	LO_2_* + LH → LO_2_H + L*	1.9 × 10	L mol^−1^ s^−1^	
R9	L* + L* → NRS_1_	6.6 × 10^4^	L mol^−1^ s^−1^	
R10	L* + LO_2_* → NRS_2_	1.0 × 10^5^	L mol^−1^ s^−1^	
R11	LO_2_* + LO_2_* → NRS_3_	6.6 × 10^4^	L mol^−1^ s^−1^	

Cell disrupted scheme

3 $$Y_{{{\text{OH}}/{\text{X}}}} {\text{OH}} \cdot + \, X \to Y_{{{\text{L}}/{\text{X}}}} {\text{LH }} + {\text{DX}}\; \left( {{\text{rate constant}} = k_{d} } \right)$$

4 $${\text{OH}} \cdot + {\text{ LH}} \to {\text{L}} \cdot + {\text{ H}}_{ 2} {\text{O}}\;\left( {{\text{rate constant}} = \, k_{\text{Lp}} } \right)$$

where *Y*_OH/X_ is the OH* needed to disrupt 1 mol of biomass, *Y*_L/X_ is the coefficient for amount (mol) of lipid released when 1 mol of biomass cell has been disrupted (amount of lipid peroxidation), DX is the disrupted cell, LH is the unsaturated lipid, *X* is the microalgae cells (biomass).

The radical reaction chain and peroxidation of lipid molecules were performed according to the initial, propagation and termination steps. The rate constants for the reaction steps are found in Table 3 and the reaction chain was described for the key reactions in the cell disruption from the interaction of radical species and lipid molecules.

Initiation (Homolytic Reaction)

5 $${\text{H}}_{ 2} {\text{O}}_{ 2 } \to 2 {\text{OH}} \cdot$$

Propagation

6 $${\text{H}}_{ 2} {\text{O}}_{ 2} + {\text{ OH}} \cdot \to {\text{HO}}_{ 2} \cdot + {\text{ H}}_{ 2} {\text{O}}$$

7 $${\text{L}} \cdot + {\text{ O}}_{ 2} \to {\text{LOO}} \cdot \, \left( {\text{lipid peroxyl radical}} \right)$$

8 $${\text{LOO}} \cdot + {\text{ LH}} \to {\text{LOOH }} + {\text{ L}} \cdot$$

Termination

9 $${\text{L}} \cdot + {\text{ L}} \cdot \to {\text{NRS}}_{ 1}$$

10 $${\text{LOO}} \cdot + {\text{ L}} \cdot \to {\text{NRS}}_{ 2}$$

11 $${\text{LO}}_{ 2} \cdot + {\text{ LO}}_{ 2} \cdot \to {\text{NRS}}_{ 3}$$

The hydroxyl radical OH* is highly reactive of free radical with short life span, therefore, the steady state of the concentration value for hydroxyl radical could be ignored. The concentration of the OH radical could be integrated through Eq. (12). Besides, the initial formation of hydroxyl could be described from Eq. (13) for the evolution of time and the effect of concentration of OH radical from the UV radiation from florescent lamp in normal room condition with intensity 8 μmol/m^2^/s.

12 $$\left[ {{\text{OH}}*} \right] = \frac{{k_{5} \left[ {{\text{H}}_{2} {\text{O}}_{2} } \right]}}{{k_{5} \left[ {{\text{H}}_{2} {\text{O}}_{2} } \right] + y_{{{\text{OH}}/X}} k_{d} \left[ x \right] + k_{\text{Lp}} \left[ {\text{LH}} \right]}}$$

13 $$\frac{1}{2} \frac{{{\text{d}}\left[ {{\text{H}}_{2} {\text{O}}_{2} } \right]}}{{{\text{d}}t}} = - k_{4} \left[ {{\text{OH}}*} \right]$$

14 $$\frac{{{\text{d}}\left[ {{\text{H}}_{2} {\text{O}}_{2} } \right]}}{{{\text{d}}t}} = - k_{5} \left[ {{\text{OH}}*} \right]\left[ {{\text{H}}_{2} {\text{O}}_{2} } \right].$$

The formation of hydroxyl radical could be estimated from differentiating equation from Eq. (14) through ordinary differential equation (ODE) from Matlab 2018a to express the radical formation time taken and concentration. Initial condition [*x*] = 0.8229 mol/L (experimental value for 20 mg of biomass for conversion), [${\text{H}}_{2} {\text{O}}_{2} ]$ = 0.03837 mol/L, [LH] = 0.03837 mol/L (assumption value for every H_2_O_2_ reacted with lipid and formed lipid hydroxide).

## Result and discussion

### Effect of concentration of hydrogen peroxide toward lipid extraction

The concentration of hydrogen peroxide in the hybrid liquid biphasic system (HLBS) will directly affect the yield of lipids. Formation of hydroxyl or peroxyl is through homolytic reaction, where the bond breaking from the molecules creates abnormal valency that causes the radicals to seek electrons to substitute the valency [37]. The radical molecules may attract the electron from the cell wall surface of the microalgae biomass to form new bonds out of the cellular wall. The rapid radical formation through Fenton reaction is potential in causing disruption to the cell wall membrane of microalgae which leads to the release of nutrient compounds including lipids, making these compounds dissolve in the organic solvent phase [21]. The current method is suitable for extracting compounds in industrial applications and will not pose concerns of the radical degrading the extracted compound and causing peroxidation for lipid. This will avoid the lipid peroxidation chain reactions that leads to the formation of highly reactive electrophilic aldehydes, and hinder its negative effect for human internal organs [38, 39]. The radical formation is supported by florescent UV in open space condition with intensity of 8 μmol/m^2^/s without any external equipment for further enhancing the homolytic reaction.

**Fig. 1:**
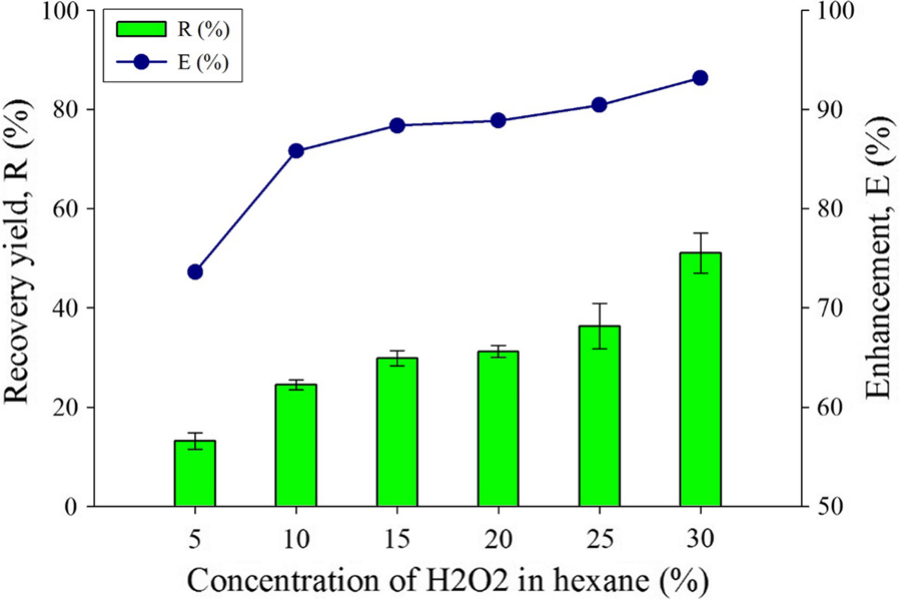
Concentration of peroxide with hexane effect for the extract lipid productivity for recovery yield and enhancement comparison toward water-organic solvent biphasic system from *C. Sorokiniana* CY-1. Other operating condition fix at 3 mL of 85% concentration of organic solvent (hexane), contact time at 1 min, biomass 20 mg (± 1 mg), and at room temperature 25 °C

Figure 1 shows the effect of hydrogen peroxide concentration on the lipid recovery yield and enhancement of the process. The highest recovery of lipid was observed at 30% concentration of hydrogen peroxide solution which yielded 50.9% lipid. The results show an increasing trend from the lowest hydrogen peroxide concentration, from 5 up to 30%. The maximum purity of hydrogen peroxide used in this work was limited to 30%, while for the purity of hydrogen peroxide, values higher than 30% would result in a highly oxidizing agent with strong oxidative properties that is usually used in the aerospace propulsive system as a hypergolic propellant thruster, also known as propulsion grade of reagent [40]. Therefore, the maximum concentration of the hydrogen peroxide does not exceed 30% in this experiment. Hydrogen peroxide is a polar solvent that attracts polar compound, while organic solvent tends to dissolve neutral or non-polar lipids [41]. Polar lipid consisting of glycolipid and phospholipid as the major components will affect the biofuel properties and conversion rate [42].

**Fig. 2:**
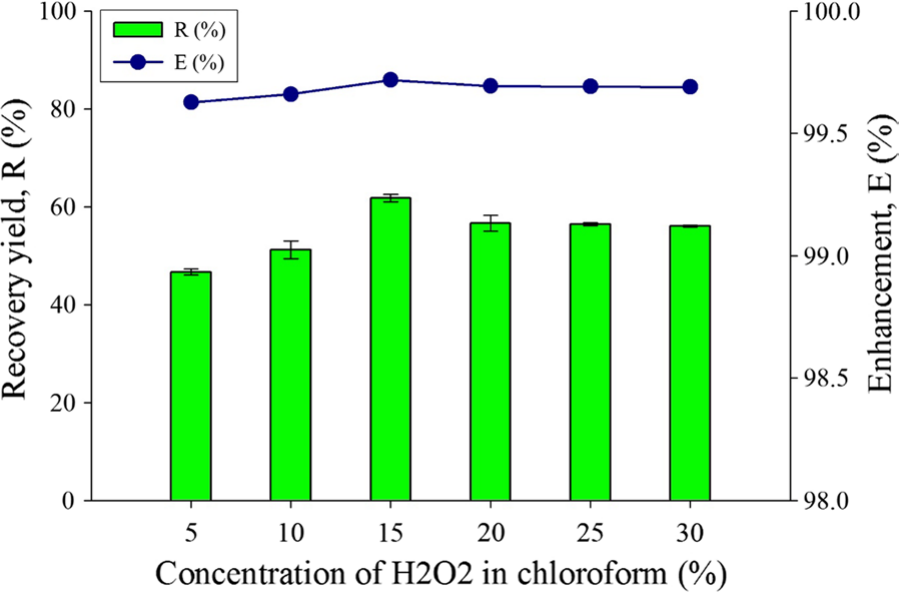
Concentration of peroxide with chloroform effect for the extract lipid productivity for recovery yield and enhancement comparison toward water-organic solvent biphasic system from *C. Sorokiniana* CY-1. Other operating condition fix at 3 mL of 85% concentration of organic solvent (chloroform), contact time at 1 min, biomass 20 mg (± 1 mg), and at room temperature 25 °C

Chloroform compared with hexane as the organic solvent, have a higher density at 1.49 g/cm^3^ compared to the density of hexane (0.65 g/cm^3^) and hydrogen peroxide (1.45 g/cm^3^). Thus, chloroform will settle to the bottom of the solution along with the biomass. Additional steps such as centrifugation were needed to fully separate the biomass and the organic solvent. Moreover, chloroform will not clearly separate the polar lipid into the surface of the solution, where it dissolves both polar lipid and non-polar lipid into the solvent. The HCl molecules may also convert the chloroform into carbon dioxide which is unwanted. Figure 2 shows the lipid recovery and enhancement using chloroform as an organic solvent with varying concentration of radical solvent for lipid extraction. The highest enhancement (99.7%) and recovery yield (61.7%) for lipid was found at 15% H_2_O_2_ in chloroform. Comparing similar concentrations at 30%, the use of chloroform combination showed about 5% higher in recovery yield compared to the hexane combination.

The hydrogen peroxide concentration in chloroform at 5% have the lowest recovery yield of 46.65% and enhancement of 99.63%, while the highest lipid recovery yield and enhancement was 61.8% and 99.7%, respectively at 15% concentration of hydrogen peroxide in chloroform. The overall chloroform mixture results for recovery yield is higher than hexane as chloroform dissolves both polar and non-polar lipids in the solvent, where it will add salt solution in the solvent for washing and purification purposes [30]. The suitable type of lipid-based biofuel conversion utilizes neutral lipid which can perform better during the conversion into biofuel. Chloroform is tautomeric in-equilibrium where there is common relocation of the proton in the molecules. During light beam lighting on electrons, the tautomeric effect causes absence of energy returning electrons back to the ground state, therefore creating no energy dissipation and causing unstable measurements in the UV–Vis reading for chloroform [43]. Due to the characteristic of chloroform which dissolves both the polar and non-polar lipids, these lipids are not favorable to be used as biofuels because polar lipid from phosphorous and glycolipid affects the combustion and causes environmental issues. Furthermore, the tautomeric and aprotic effect does not contribute to better stability of the biofuel and may disturb the spectroscopic analysis performance. Therefore, hexane was selected as the better solvent of the two for lipid extraction.

### Effect of solvent concentrations

**Fig. 3:**
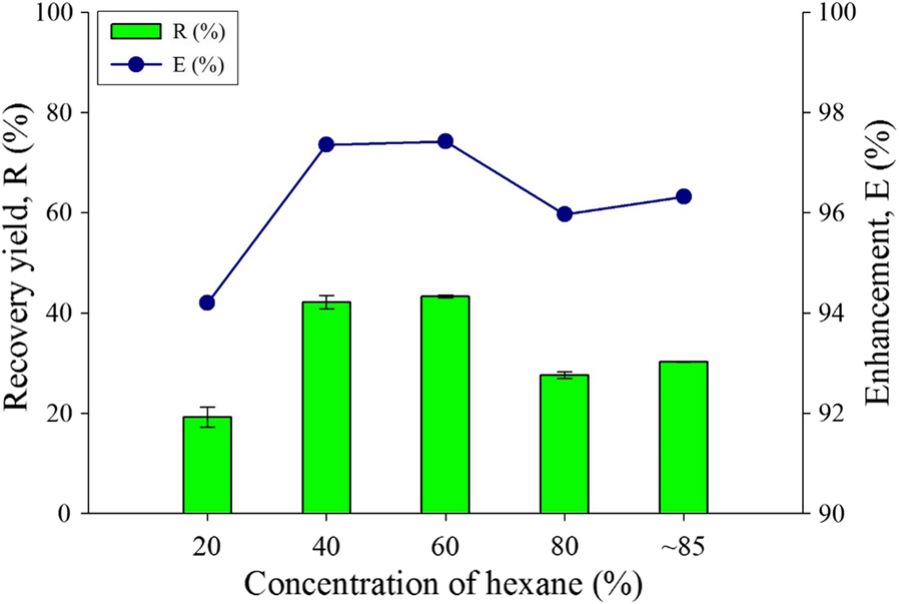
Concentration of organic solvent (hexane) effect for the extract lipid productivity for recovery yield and enhancement comparison toward water-organic solvent biphasic system from *C. Sorokiniana* CY-1. Other operating condition fix at 3 mL of 30% concentration of hydrogen peroxide, contact time at 1 min, biomass 20 mg (± 1 mg), and at room temperature 25 °C

Organic solvents play a vital role for lipid extraction due to its capability of dissolving lipids. The concentration for organic solvents in the HLBS has been altered to observe the effect towards recovery yield and enhancement. The concentration of organic solvent was investigated in the range of 20–85%. From Fig. 3, the optimum concentration for hexane is 60% for *R* and *E* value which is 43.2% and 97.42% respectively. The dilution of hexane is done with acetone which is nonreactive, non-metabolized, low viscosity and able to mixed well with most organic solvents [44]. The least recovered yield of lipid (19.17%) is at 20% concentration of hexane. The highest purity 85% of hexane showed reduced recovery yield at 30.2% and the enhancement at 96.3% compare to 60% concentration of hexane dilution with 40% of acetone. The highest recovery yield and enhancement for hexane dilution is at 60% where 97.4% of enhancement and 43.2% of recovery yield was achieved. Acetone can dissolve in polar and non-polar solvents, it consists of non-polar methyl groups and polar carbonyl (C=O) groups that allows it to dissolve in most polar and non-polar solvent types. Acetone is also an aprotic solvent with polar features and is prone to attract polar compound such as phospholipid and glycolipid [45]. Hence, acetone was selected as the suitable choice for its capability to dissolve in most types of non-polar solvent including chloroform and hexane without forming two different phases.

**Fig. 4:**
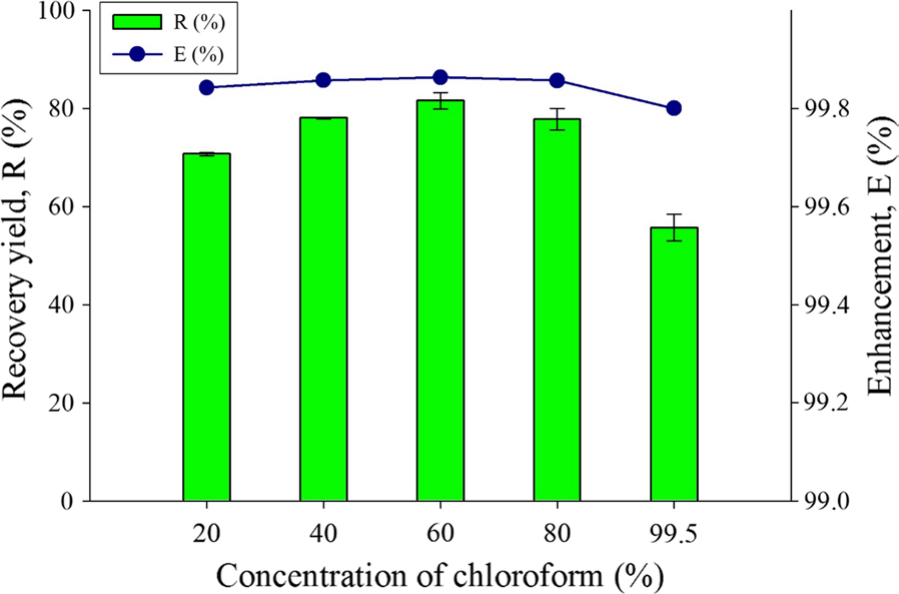
Concentration of organic solvent (chloroform) effect for the extract lipid productivity for recovery yield and enhancement comparison toward water-organic solvent biphasic system from *C. Sorokiniana* CY-1. Other operating condition fix at 3 mL of 30% concentration of hydrogen peroxide, contact time at 1 min, biomass 20 mg (± 1 mg), and at room temperature 25 °C

The concentration varying for chloroform for extracting lipid was shown in Fig. 4, the optimum concentration for chloroform is at 60% and the enhancement is at 99.8% while recovery yield is 81.5%. The pure chloroform shown 99.8% enhancement and 55.7% recovery yield which is significantly lower for by 25.8% compare to 60% concentration of chloroform, this indicates that too high chloroform concentration will greatly reduce the lipid recovery yield. The optimum result for dilution of organic solvent for lipid extraction was selected to be 60% dilution of chloroform solvent with 40% of acetone.

Comparing with hexane dilution parameter, both the enhancement and recovery yield of using chloroform are higher. However, chloroform and acetone dilution for optimizing the lipid content is unstable due to the aprotic and tautomeric effect. According to the eluotropic series, taking silica as stationary medium indicates that hexane is a non-polar solvent with low detecting value in UV–Vis compared to chloroform [46, 47]. This means that the minimum detecting value for hexane is lower compared to most organic solvents including chloroform, acetone and toluene. Moreover, hexane is a favorable choice for solvent as it has higher stability and is more accurate for the parameter determination. It is reported that there are more monounsaturated fatty acids than polyunsaturated fatty acids in extraction processes using hexane derived solvents [48]. The monounsaturated fatty acids are preferred as the quality components for biodiesel properties in terms of low temperature fluidity and oxidative stability [49]. Therefore, hexane of 60% is been selected as the choice of organic solvent with 43.2% of lipid recovered yield.

### Effect of different ratio (H_2_O_2_:Hexane)

**Fig. 5:**
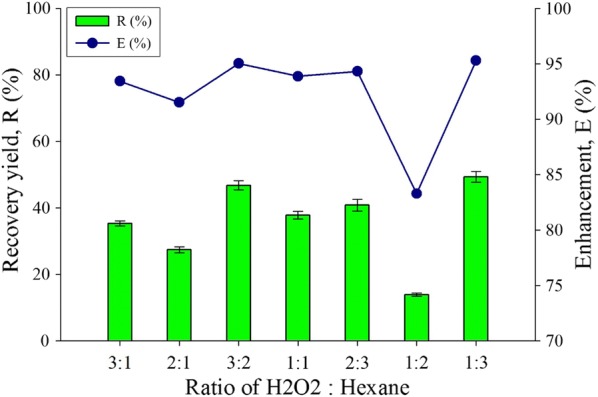
Ratio of (H_2_O_2_: Hexane) effect for the extract lipid productivity for recovery yield and enhancement comparison toward water-organic solvent biphasic system from *C. Sorokiniana* CY-1. Other operating condition fix at 30% concentration of hydrogen peroxide, pure analytical grade of organic solvent (hexane), contact time at 1 min, biomass 20 mg (± 1 mg), and at room temperature 25 °C

Due to tautomeric effect and aprotic solvent distraction, pure hexane is chosen for optimizing this parameter for enhancing the lipid extraction for HLBS system. In Fig. 5, cell disruption and organic solvent extraction ratio in the system was altered for the lipid productivity of microalgae biomass. In the HLBS, the bottom aqueous layer consists of solvents for cell disruption of the biomass and the top organic layer contains hexane for dissolving lipids. The ratio for both solvents was studied to evaluate the effect of alternating the ratio of the solutions on the enhancement and recovery rate. The optimum ratio for lipid extraction is 1:3 where the *E* and *R* value are at 95.3% and 49.3% respectively. By increasing the cell disruption phase, the recovery yield does not increase proportionally, as the recovery yield for ratio 1:1, 2:1 and 3:1 showed 37.8%, 27.4% and 35.3%, respectively. Whereas by adjusting the ratio for cell disruption and organic phase will lead to a significant increase in recovery by about 3.5-fold when compared between the ratio 1:2 and 1:3. For a constant H_2_O_2_ amount, adding more organic solvent will assist in dissolving more lipids from the biomass.

H_2_O_2_ ratio in the default time of contact of 1 min accumulated more lipid from the biomass compared to 1:2 (H_2_O_2_:Hexane). This may be due to more peroxyl and hydroxyl radicals from the H_2_O_2_ assisting in the cell disruption once the H_2_O_2_ ratio has increased. The introduction of H_2_O_2_ assures the presence of peroxyl and hydroxyl radicals in the solvent to cause disruption of the cell membrane and thus allowing lipid molecules to dissolve into the organic solvent. The contact time represents the cycle of radical propagation frequency of the biomass in HLBS system, which plays a major role for preventing further disruption of the lipid compounds [50]. This parameter evaluation has indicated that the higher organic solvent with a same disruptive solvent amount will result in higher recovery of lipids as there will be more available volume for the lipids to dissolve into the system.

### Effect of volume differences

**Fig. 6:**
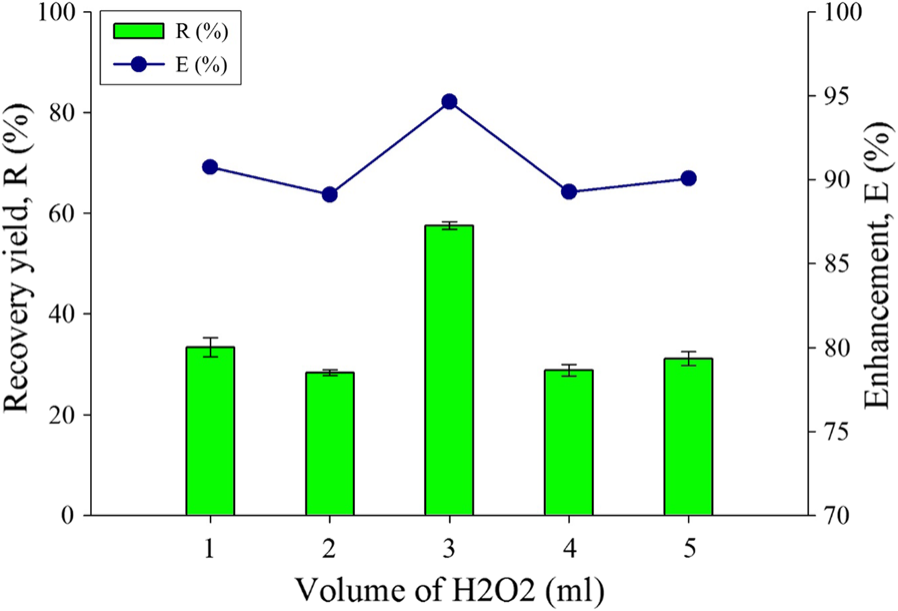
Volume of hydrogen peroxide (H_2_O_2_) effect for the extract lipid productivity for recovery yield and enhancement comparison toward water-organic solvent biphasic system from *C. Sorokiniana* CY-1. Other operating condition fix at 30% concentration of hydrogen peroxide, pure analytical grade of organic solvent 85% (hexane) at volume 3 mL, contact time at 1 min, biomass 20 mg (± 1 mg), and at room temperature 25 °C

The difference in volume for the H_2_O_2_ cell disruption layer was studied and its effect on the lipid recovery yield and enhancement is shown in Fig. 6. The highest extracted lipid is with 3 ml of volume, obtaining 94.6% enhancement and 57.5% of recovery yield of lipid. The addition of more hydrogen peroxide volume into the HLBS system may tend to induce more hydroxyl and peroxyl radicals which should increase the cell disruption rate [51], however, the results shows an inconsistent trend. Too much radicals may have cause the disruption of the lipids which result in lower recovery, hence it is not advantageous to add a higher amount of cell disruptive solvent. The least recovery and enhancement is in 2 ml of hydrogen peroxide, which is at 28.37% and 89.1% respectively. The decreasing cell disruption layer phase volume in general does not contribute to a significant recovery yield of lipid. This is due to the cell membrane properties which needs more radical propagation cycles to disrupt the cell membrane of microalgae. The optimum volume amount is 3 ml as it achieved the highest yield in this experimental parameter.

**Fig. 7:**
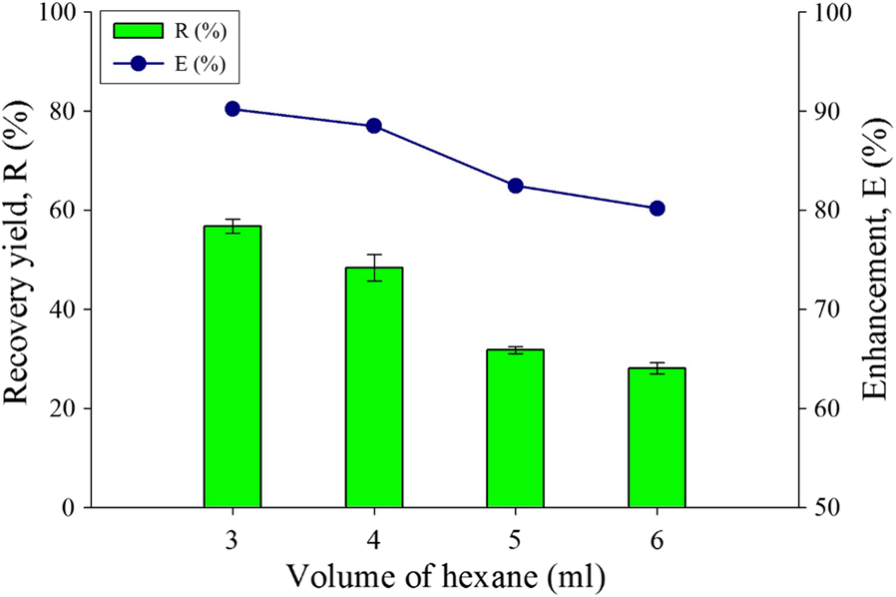
Volume of organic solvent (hexane) effect for the extract lipid productivity for recovery yield and enhancement comparison toward water-organic solvent biphasic system from *C. Sorokiniana* CY-1. Other operating condition fix at 30% concentration of hydrogen peroxide at volume 3 mL, pure analytical grade of organic solvent 85% (hexane), contact time at 1 min, biomass 20 mg (± 1 mg), and at room temperature 25 °C

The volume difference of organic solvent in the HLBS system was also studied and the results of lipid recovery and enhancement are shown in Fig. 7. The highest extracted lipid amount is at 3 ml of hexane and 1 ml of hydrogen peroxide in the HLBS system, achieving an enhancement of 90.2% and recovery yield of 56.7%. A decreasing trend was observed by adding more volume of organic solvent (hexane) into the HLBS system. Hexane has a delta value of 14.9 δ for total and partial solubility value after propane in the non-polar fluids categories, indicating the solubility value for oil and lipid [52]. Thus, by adding more solvents of the same solubility into the HLBS system will not enhance the extraction capability but may dilute the solution for analysis of lipid compound. The enhancement in Fig. 7 shows a decrement by more than 10% as more hexane were added in the system. The optimum volume for HLBS is 3 mL in this experimental parameter.

### Effect of contact time

**Fig. 8:**
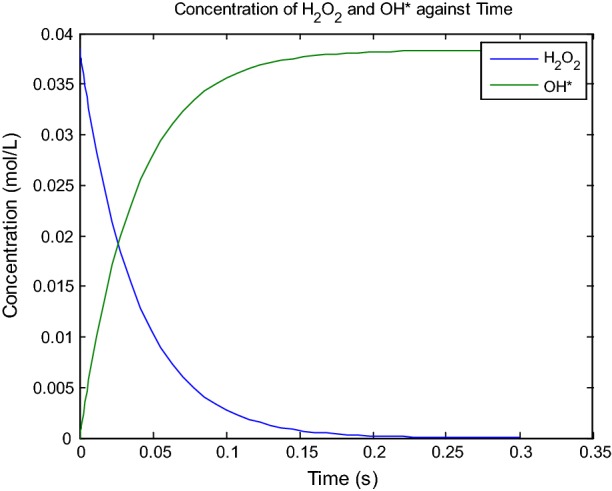
The graph of concentration for H_2_O_2_ and hydroxyl radical formation against time simulate by MATLAB 2018a. The concentration of hydrogen peroxide 30% in 2 mL, in room temperature condition 25 °C, with the presence of florescent lamp at intensity 8 μmol/m/s

**Fig. 9:**
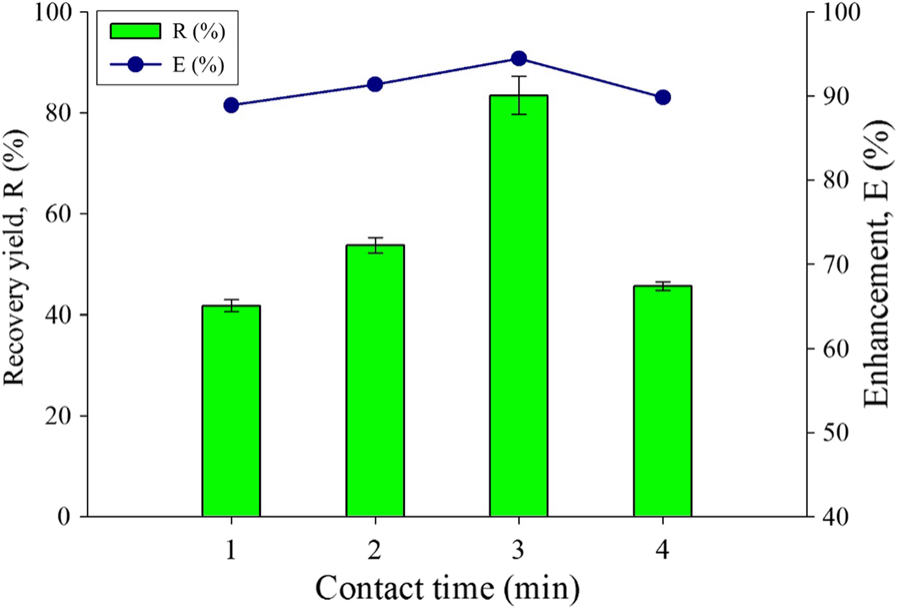
Time of contact effect for the extract lipid productivity for recovery yield and enhancement comparison toward water-organic solvent biphasic system from *C. Sorokiniana* CY-1. Other operating condition fix at 30% concentration of hydrogen peroxide, pure analytical grade of organic solvent 85% (hexane), biomass 20 mg (± 3 mg), and at room temperature 25 °C

The concentration of hydrogen peroxide and hydroxyl radical formation against time was simulated by using the mathematical modelling, where the time of hydroxyl radical formation was completed within 0.35 s. The estimated concentration was at 0.035 mol/L for the hydroxyl radical (Fig. 8). The optimized time may enhance the lipid extraction into the organic solvent. This parameter may affect the degradation rate of the cell membrane with the initial reasoning that longer contact time of the cell disruption phase layer could improve the productivity of lipid extraction. The highest lipid recovery yield and enhancement is with 3 min contact time at 83.5% and 94.4% respectively (Fig. 9). The least enhancement and recover yield are found within 1 min of contact time at 88.9% and 41.7% respectively. According to the study by Concas et al. the peak value of time contact exceeding 3 min will reduce lipid production and our results are in accordance with their work [21]. The recovery yield and enhancement of lipid extraction reached the peak at 3 min and started fall at 4 min. The time of contact may increase the cell disruption and lipid will be released more efficiently in the organic solvent, resulting in more lipids accumulation in the solvent. Prolonged contact time will eventually caused the disrupiton of lipids that leads to lower lipids production. The radical cell disruption method has also been reported to enhance the quality of biodiesel, as the characteristic of biodiesel produced with radical disruption method are more stable with reduction of undesired polyunsaturated fatty acid, such as linolenic acids (C18:3), from the lipid molecular structure [53]. Furthermore, the lipids produced may be used as industrial intermediate products for polymer crosslinking products, since the lipid compounds extracted have been radically treated and retain a partially functionalized form of the unsaturated chains from the hydrocarbon molecules [54]. The contact time of 3 min showed the best *R* and *E* and hence it was selected as the optimum value for this experimental parameter.

### Effect of biomass amount with solvent ratio and GC-FID lipid profile

The retention time for the lipid profile comparison in Table 4 has shown that the percentage error for peak 1, 2, 3, and 4 are less than 1%. The similarity of the lipid profile through GC-FID results for these 4 peak components in Table 4 is apparent and observed with the lipid extraction in the optimized HLBS solution mixture. The results from GC-FID indicates that the lipids extracted are comparable to that of commercial triglyceride and the HLBS process used is very potential in recovering the lipids efficiently. In chromatography, the detect signal for the analyte is directly proportional to the concentration of the component in the solution against functional of time [55]. These components of fatty acids are the potential tail which are attached to the glycerol to form triglycerides in lipids.

**Table 4:** Comparison of lipid profile using GC-FID

Peak	HLBS (mins)	Pure triglyceride mixture (mins)	Area of graph for HLBS	Percentage of error (%)
1	1.663	1.664	42.94	0.06
2	1.859	1.861	28.62	0.11
3	2.097	2.1	23.4	0.14
4	2.411	2.416	5.04	0.21

The effect of biomass amount correlates with the ratio of solvent in HLBS for improving the lipid productivity. Increase in biomass amount will increase the lipid output from the final extraction process, but it will not directly improve the lipid recovery yield and enhancement value (Table 5). The optimum ratio for 20 mg of biomass was found at 1:3 (H_2_O_2_:Hexane solvent ratio), where the values *E* and *R* achieved 93.8% and 37.4% respectively. In 30 mg of biomass, the 2:3 solvent ratio is best where the *E* and *R* values achieved 93.1% and 33.6% respectively. The value for 40 mg of biomass achieved higher productivity at 2:3 ratio, where the recovery is 53.9% and enhancement is 95.7%. While for 50 mg of biomass, 2:3 was the optimum ratio for extraction as the *R* and *E* values reported were the highest. The addition of more biomass does not assist in lipid recovery, but it demonstrates a relationship between varying biomass weight with ratio which will affect the lipid recovery and enhancement value. This is due to more contact surface area for cell disruption when more biomass is added within the same amount of solvent. Ratio of 1:3 showed higher lipid recovery yield at 54.6% with 50 mg of biomass and enhancement value of 95.8%. Besides, for ratio 2:3 the highest lipid recovery and enhancement is at 55.1% and 95.8%, respectively, which showed improvements of 1.2% for *R* and 0.09% for *E* value compare to 40 mg with the same ratio. The ratio for 3:3 have the highest *E* and *R* value at 50 mg which are 94.9% and 45.4% respectively, while the value is higher than 3:4 ratio with the same amount of biomass at 50 mg with an increment of 1.26% in *E* and 9% in *R* value. Ratio 2:3 (H_2_O_2_:Hexane) is the most versatile ratio to various biomass amount in this experimental parameter. It is deduced that a suitable ratio of the solvents will assist in optimizing the extraction through reducing chemical solvent consumption as well as lowering the material costs needed.

**Table 5:** The effect of biomass with different ratio for the extraction of lipid for recovery yield and enhancement comparison toward water-organic solvent biphasic system from *C. Sorokiniana* CY-1

Ratio	1:3		2:3		3:4		3:3	
Amount (mg)	*E%*	*R%*	*E%*	*R%*	*E%*	*R%*	*E%*	*R%*
20	93.8	37.4	89.4	21.9	87.6	18.7	88.7	20.7
30	93.0	33.2	93.1	33.6	88.7	20.6	89.7	22.5
40	93.5	35.5	95.7	53.9	93.4	35.5	91.3	26.8
50	95.8	54.6	95.8	55.1	93.6	36.4	94.8	45.4

Other operating condition fixed at 30% concentration of hydrogen peroxide, organic solvent 85% (hexane), and at room temperature 25 °C

## Conclusions

The hybrid liquid biphasic system (HLBS) in a single solution with two different phases in the system is efficient for the extraction of lipids from microalgae. The combination of cell disruption pre-treatment along with simultaneous extraction process will contribute to cost and energy saving by reducing the multistep procedure to a single-step extraction technique. Furthermore, this HLBS method could be applied in producing biofuels with enhanced properties through treatment in the hybrid system. Both the extraction and cell disruption will occur in two immiscible phases for ease of separation which is sustainable as there is potential to recycle of the solvents in large scale applications. More than 80% of lipids were successfully recovered from microalgae biomass and the extraction enhancement compare to water biphasic system achieved was higher than 90%. This hybrid biphasic system is potential to be applied in lipids extraction involving biomass with thick cell wall, especially for commercial biofuel production which requires biofuels of enhanced quality performance.

## Data Availability

Not applicable.
